# Employment disadvantage and associated factors for informal carers of adults with mental illness: are they like other disability carers?

**DOI:** 10.1186/s12889-019-6822-1

**Published:** 2019-05-16

**Authors:** Sandra Diminic, Emily Hielscher, Meredith G. Harris

**Affiliations:** 1 School of Public Health, Faculty of Medicine, The University of Queensland, Brisbane, Australia; 2 Policy and Epidemiology Group, Queensland Centre for Mental Health Research, The Park - Centre for Mental Health, Locked Bag 500, Archerfield QLD, Brisbane, 4108 Australia; 3 Centre for Clinical Research, The University of Queensland, Brisbane, Australia

**Keywords:** Australia, Caregivers, Informal care, Mental disorders, Employment, Labour force

## Abstract

**Background:**

Providing unpaid support to family and friends with disabling health conditions can limit a carer’s capacity to participate in employment. The emotional support needs and unpredictability of caring for people with mental illness may be particularly demanding. While previous research suggests variable employment rates across carers for different conditions, there are limited data on mental health carers specifically.

**Methods:**

This study analysed employment patterns for working-age, co-resident carers of adults with mental illness in an Australian cross-sectional household survey, the 2015 Survey of Disability, Ageing and Carers.

**Results:**

Significantly more mental health carers were not employed (42.3%, 95% CI: 36.6–48.1) compared to non-carers (24.0%, 95% CI: 23.5–24.6). Employed mental health carers were more likely to work fewer than 16 h per week (carers: 17.2%, 95% CI: 12.8–22.8, vs. non-carers: 11.7%, 95% CI: 11.3–12.1) and in lower skilled occupations (carers: 22.6, 95% CI: 17.5–28.7, vs. non-carers: 15.7, 95% CI: 15.1–16.2). Among the sub-group of primary mental health carers, 25.8% (95% CI: 15.6–39.5) had reduced their working hours to care and a further 26.4% (95% CI: 17.2–38.2) stopped working altogether. In corresponding comparisons between mental health carers and carers for people with other cognitive/behavioural conditions, and physical conditions with or without secondary mental illness, there were no differences except that mental health carers were more likely to be working in a lower skilled occupation than other cognitive/behavioural condition carers (14.8% of the latter, 95% CI 10.1–21.2). Multivariate logistic regression analyses revealed that female mental health carers were less likely to be employed if they were aged 35–54, had no post-secondary education, had a disability, or cared for someone with severe activity limitations. For male mental health carers, having a disability or caring for someone with severe limitations or who did not receive paid assistance were significantly associated with not being employed.

**Conclusions:**

These results highlight the employment disadvantage experienced by mental health carers compared to non-carers, and similarities in employment patterns across carers for different conditions. Improving the availability of paid support services for people with mental illness may be an important target to assist carers to maintain their own employment.

**Electronic supplementary material:**

The online version of this article (10.1186/s12889-019-6822-1) contains supplementary material, which is available to authorized users.

## Background

Family, friends or neighbours of people with long-term health conditions and disabilities often take on the role of an informal carer, providing assistance with a range of practical and support tasks. Cross-sectional and longitudinal studies demonstrate that carers are less likely to be employed than non-carers [1], with the main effect on employment status or labour force participation rather than hours worked [2]. For working carers, juggling the competing demands of both intensive caring and employment can be stressful and exhausting [3, 4]. Carers may reduce their working hours, take more leave, exit the labour force altogether, or make other adjustments to accommodate caring, such as choosing a more flexible or conveniently located job associated with a less challenging role or poorer remuneration [5, 6]. Carers’ participation in employment is also associated with other characteristics of the carer—such as middle age, being male, and in better health; the person they care for—such as a lower level of disability; and the caring role—such as lower intensity of caregiving [1, 6]. However, there has been comparatively little research exploring the relationship between caring for people with mental illness (e.g. psychotic, anxiety, depressive or personality disorders) and employment, despite evidence that the caring role in these circumstances is quite different [7].

Mental health caring differs from caring for people with other conditions, particularly physical disabilities. It has a greater focus on emotional support, managing crises and supervision of behaviour, and often involves unexpected fluctuations in support needs associated with the episodic nature of mental illness, as well as significant amounts of time spent ‘on call’ in case a crisis occurs [8, 9]. Mental health carers tend to report a higher caring burden and greater unmet support needs than carers of people with physical conditions [9, 10]. It has been argued that this emotional and crisis-related caring places additional stress on carers and interferes more with paid employment than other types of care [11], although comparative evidence is lacking. Beyond the practical challenges of juggling caring and work tasks, mental health carers report significant anxiety and poor health associated with their caring, which in turn impacts their work performance [11]. However, the workplace may also provide a form of respite for struggling mental health carers, similar to patterns described for emotionally strained dementia carers [12]. Further, mental illnesses have a younger age of onset than many other conditions, such as cardiovascular, musculoskeletal and neurological disorders [13]. Mental health caring therefore affects individuals at a broader range of ages and life stages, and may be long-term [10, 14]; this is important for its impact on carers’ employment [15].

Previous studies suggest varying employment rates across carers for different conditions, but findings have been mixed. One Australian study using the 2009 Survey of Disability, Ageing and Carers (SDAC) found that more than 60% of primary carers for some of the 25 conditions—like acquired brain injury, schizophrenia and osteoporosis—were not in the labour force, compared to less than 40% for others—such as attention deficit disorder and arm/hand/shoulder injuries [16]. An analysis of the 2012 US National Health and Wellness Survey found no difference in employment between schizophrenia carers, other carers (including for bipolar disorder and dementia) and non-carers, although working schizophrenia carers reported higher absenteeism, presenteeism and overall burden [17]. However, carer groups were matched on household income, which may have limited between-group differences in employment. A large-scale sample from the 2009–10 Personal Social Services Survey of Adult Carers in England found no difference in employment rates between carers for individuals whose conditions included or did not include a mental health problem, learning disability or dementia [18]. Drawing consistent conclusions from these diverse findings is challenging, and further complicated by differing labour market conditions, health care services and support arrangements for carers across countries [1, 19].

In Australia, there has been limited quantitative research to guide efforts to support mental health carers in the workforce. Our recent analysis of the 2012 SDAC found only 53.5% of mental health carers were employed, while for primary mental health carers (i.e. the person providing the most support), the figure was even lower at 40.8% [9]. An earlier survey of mental health carers receiving a caring pension (Carer Payment) or supplementary financial assistance (Carer Allowance) from the Australian government showed that only 29 and 53% respectively were employed [11]. Many of these carers reported making other work accommodations due to their caring, such as not applying for jobs (45%), reducing working hours (44%), or changing to a role with less responsibility and pay (25%) [11]. Other studies including smaller samples of Australian mental health carers recruited through health services also report that less than half are employed [20, 21]. In comparison, 62% of the Australian population were employed in late 2017 [22]. These studies illustrate the apparent low employment rates of Australian mental health carers but do not provide direct comparisons with other carers or the general population.

There is a good economic and social rationale for supporting carers to maintain their employment. For the carer, time out of the workforce leads to lost income, disruption to their career trajectory, and potentially other negative effects of unemployment such as reduced social networks and poorer health [23]. Conversely, employment has been linked to better mental health and quality of life for carers [24, 25]. From a government perspective, the costs of inaction include lost tax revenue from employed carers’ earnings, increased costs to provide income support to some carers (e.g. Carer Payment), and lost productivity and return on investment in education and training when skilled workers reduce their hours or leave the workforce [26–28]. Where unemployment contributes to poorer health for carers, there may also be increased health and support service costs to government. Recognising these issues, Australia’s Fifth National Mental Health and Suicide Prevention Plan [29] includes an indicator for the proportion of mental health carers in employment.

To guide supports for mental health carers’ employment, specific information is needed about the factors most closely associated with employment, particularly those that may be amenable to policy intervention. Research conducted internationally on all disability carers has consistently identified that carers are less likely to be working if they are: female; nearing retirement age; less educated; in poorer health; or have a higher caring intensity, including greater caring hours, being the primary informal caregiver, caring for a close relative, living with the person they support, and caring for more than one person or someone who is more disabled [1, 6, 30]. Additional factors in some studies include the carer’s ethnicity or country of origin [31], marital status [30, 32, 33] and whether the person they care for receives disability support services [6, 18]. Use of paid services by the person with disability has been positively associated with employment for UK carers providing 10 or more hours of care weekly [18]. The relative importance of these contributing factors varies between male and female carers [2, 18, 34]. However, it is not known which variables are most important for mental health carers, who may be at different life stages and have access to a different range of health and support services compared to other carers. This study aimed to explore potential employment disadvantage experienced by mental health carers and the factors associated with their employment.

## Methods

### Aims

This study analysed a pre-collected, nationally representative household survey on disability and informal caring. The aims were to identify: (1) whether co-resident mental health carers are more disadvantaged in employment than carers of people with other types of disabilities and non-carers; (2) which factors are most strongly associated with employment for mental health carers; and (3) whether there are unique factors associated with mental health carers’ employment compared to carers for other conditions.

### Survey and sample

The 2015 SDAC [35] was a nationally representative household survey carried out by the Australian Bureau of Statistics (ABS) between July and December 2015. Households were selected from a stratified, multi-stage area sample developed by the ABS. Basic demographic data on all household members were collected from a responsible adult in each household (i.e., the first adult with whom the interviewer made contact who was able to participate), by trained interviewers using a Computer-Assisted Personal Interview. The responsible adult also answered questions to identify the presence of a person with disability or carer in the household and, where possible, additional interviews were completed with persons with disability and confirmed primary carers (but not other carers) at the same time or a later date within the survey period. Proxy interviews were conducted for people unable to be interviewed due to language or impairment, children aged below 15 years, and people aged 15–17 years without parental consent to participate. The final household sample included 25,806 households comprising 63,515 persons (80.0% response rate).

### Key variables

#### Persons with disability

Persons with a disability were identified by the responsible adult (e.g. “Does anyone in the household have a [nervous or emotional condition] that has lasted, or is likely to last for 6 months or more?”, “Are they restricted in everyday activities because of this condition?”, “Is anyone in the household receiving treatment or medication for any long-term conditions or ailments?”). Household members identified as having a disability were interviewed and provided additional information on: their main disabling condition; all conditions; level of activity limitations; and receipt of formal assistance (services) for their disability.

#### Informal carers

Carers were identified by the responsible adult (e.g. “Does anyone in the household help or supervise [another member of the household]/ [someone living elsewhere] who has a long-term health condition or disability with everyday types of activities?”, “Do they provide this help on a regular, unpaid, informal basis?”). If not initially identified by the responsible adult, carers could also be subsequently identified by a person with disability living in the household during their personal interview (e.g. “Have you received, or do you expect to receive, assistance to help with these tasks from a partner or spouse/parent, family, friends or neighbours for 6 months or more?”). The 2015 SDAC classified household members as carers where they provided support to someone with a limitation to their mobility, communication or self-care and this support was ongoing, or likely to be ongoing, for at least six months. The responsible adult (or person with disability) initially described the relationship of the carer to the person cared for and the number of people supported; carers were only asked to complete a personal interview if they confirmed that they were the primary carer of a person with disability. The 2015 SDAC identified confirmed primary carers, a subset of all carers in the survey, as the person providing the most assistance to someone with a disability. Confirmed primary carers aged 15 years or more were interviewed separately to collect additional information, including questions about the impact of their caring on employment and working hours.

This study focused on both primary and secondary carers aged 15–64 years to align with the youngest age for Australians commencing employment and aged pension eligibility, after which workforce participation drops significantly. Information on the disabling conditions of care recipients was only available for carers living in the same household, so the analysis was limited to co-resident primary and secondary carers. Four carer groups were created based on the main disabling condition of the person cared for: mental illness (e.g. psychosis, depression, anxiety, personality and behavioural disorders; *n* = 520); other cognitive/behavioural conditions (e.g. dementia, autism, intellectual disability, acquired brain injury; *n* = 312); and physical conditions (e.g. musculoskeletal, cardiovascular, neurological and sensory disabilities) with or without a secondary mental illness (*n* = 577 and *n* = 1455 respectively). Additional file 1: Table S1, includes the full list of conditions. Carers for more than one person with different conditions were grouped hierarchically, in that order (i.e. mental illness first). Cognitive conditions and secondary mental illness were separately identified because the required behaviour management and fluctuating care needs were expected to have a more detrimental impact on carers’ ability to maintain employment [11]. We focused on carers of adults with disabilities; those supporting only people aged below 15 years were excluded because of the complexities in separating the effects of informal caring on employment from those of normal parenting in a cross-sectional analysis. A comparison group of non-carers included people aged 15–64 years who did not support a person with disability of any age.

#### Employment

The 2015 SDAC recorded employment data for participants aged 15 and over. The main outcome of interest for this study was employment status—whether a person is employed or not (unemployed or not in the labour force). The 2015 SDAC defined employment as engaging in economic work of one hour or more in the survey reference week. Full-time employment is permanent, temporary or casual employment of 35 h or more per week (across all jobs), or working 35 h or more during the reference week even if the person usually works fewer hours [36]. Part-time employment is working fewer than 35 h per week [36]. Persons were classified as unemployed if they were aged 15 or over and worked less than one hour in the reference week, were actively looking for work in the previous four weeks, and were also available to start work [37]. Those who were not employed indicated their main activity since last looking for work. We also examined potential indicators of underemployment in the form of hours worked and occupational category.

### Data analysis

The ABS supplied a Confidentialised Unit Record File of the 2015 SDAC (October 2016 version). Person-level, recipient-level and condition-level data files were merged to obtain estimates for all co-resident carers and their care recipients. Analyses were conducted in Stata 15 [38], using ABS-provided survey weights to account for possible selection and non-response biases, and differences between the sample and Australian population. Survey-weighted proportions described key demographic and employment characteristics of each carer group, and 95% confidence intervals (CIs) were calculated using jackknife repeated replication.

To compare mental health carers with non-carers and other disability carers on employment status, working hours and occupational group (aim 1), three simple logistic regression models were run with binary outcomes for employment status (not employed vs. employed), working hours (< 16 vs 16+ hours per week), and occupation (machinery operator, driver or labourer vs. technical and professional roles).

Factors potentially associated with employment for mental health carers were identified based on previous studies: carer age group; marital status; rurality; country of birth; highest education level; whether the carer has a disability; whether any person cared for receives formal services for their disability, and the type, frequency of and need for these services; and indicators of caring intensity—including being a confirmed primary carer, number of people cared for, caring for a close family member (spouse/partner or adult child), and caring for someone who is profoundly or severely limited in core activities [1, 6, 18, 30–32]. Education level was recorded as ‘not determined’ for 14 of 520 mental health carers and 45 of 2344 other carers; this coding was not significantly related to employment status (χ^2^(1, *N* = 2864) = 0.10, *p* = 0.75), so these carers were excluded from the multivariate regression analyses.

To identify factors associated with employment status for mental health carers (aim 2), multivariate logistic regression models were developed to calculate adjusted odds ratios (AORs) and 95% CIs. Separate models were conducted for male and female carers due to the potentially different relationships by gender [2, 18, 34]. Pairwise Cramer’s *V* associations between factors revealed moderate relationships (*V* = 0.37–0.64) between age group, marital status and caring for a partner/child, and between primary carer status and disability level of the person cared for (Additional file 1: Table S2). However, all variance inflation factors were below three, and since these factors each represented distinct constructs of interest they were retained. Unsurprisingly, receipt of formal services was highly correlated with receiving particular types of assistance, frequency of assistance and unmet need for assistance. The latter factors were entered separately into supplementary regression analyses replacing the former in each model (see notes on, Additional file 1: Tables S5-S6). All factors were initially entered into each model and a final model selected via backwards elimination until only predictors with a *p*-value of <.10 remained. This higher cut-off than the standard significance threshold of *p* < .05 was chosen to ensure no potentially important variables were excluded [39, 40].

Further logistic regression models by gender were run to identify whether any factors associated with employment were unique to mental health carers (aim 3). These models tested interactions between the disability group of the person supported, selected factors (as below), and employment status. Bivariate chi-square tests identified which factors were significantly different between mental health versus other carers (Additional file 1: Table S3). For each gender, all significant factors as well as those identified as significantly related to employment in the mental health carer models were included in the initial regression analyses as interaction terms with disability group. To minimise loss of statistical power with the addition of interaction terms, education level and disability group were converted to dichotomous variables. Models were reduced via backwards elimination as above.

## Results

### Aim 1. Employment disadvantage

#### Employment status

**Fig. 1:**
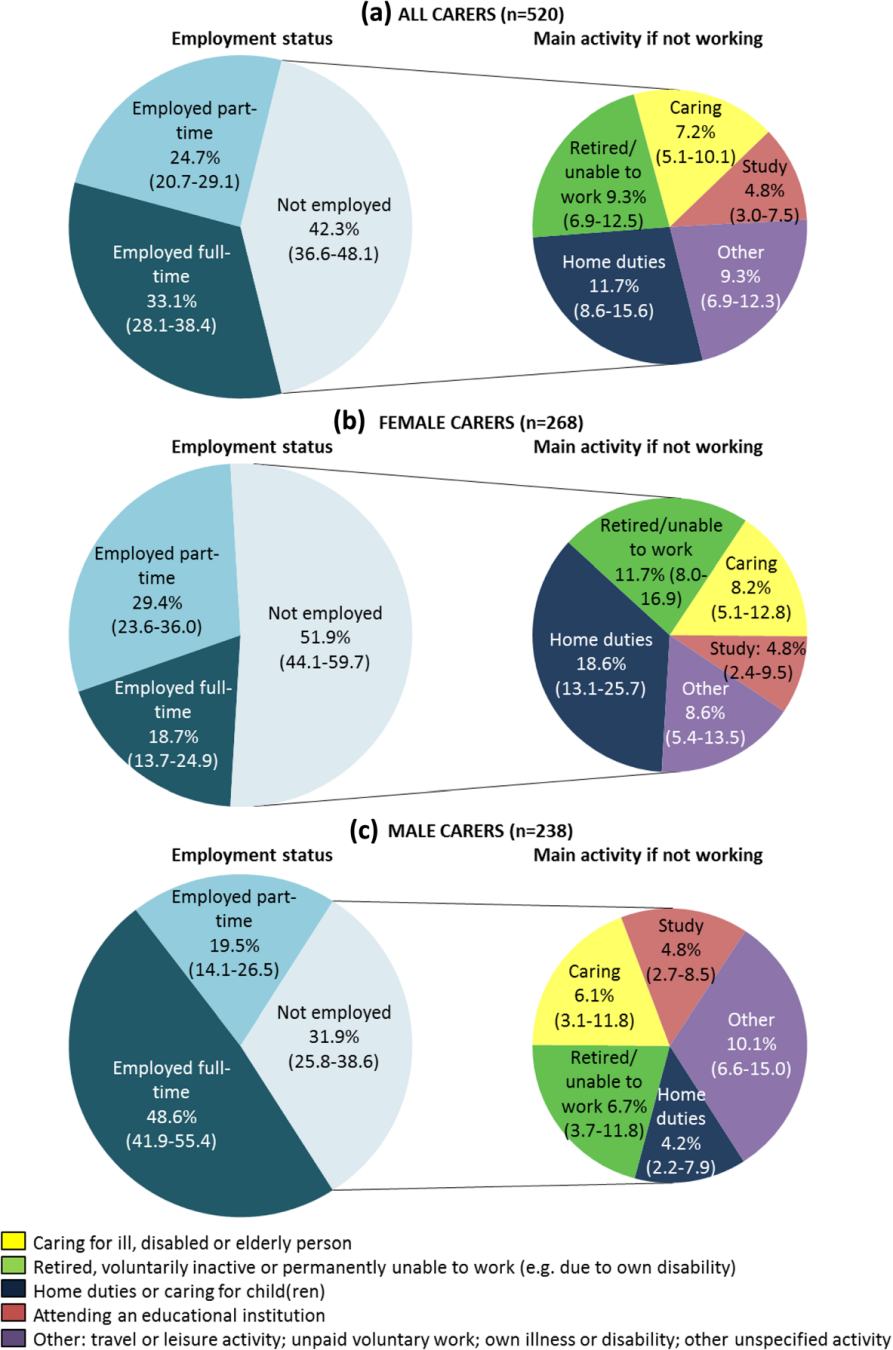
Employment status for co-resident carers aged 15–64 years of adults with mental illness, by sex. Note: numbers in brackets represent 95% confidence intervals.

In 2015, 33.1% of working-age mental health carers were employed full-time, 24.7% were employed part-time, and 42.3% were unemployed or not in the labour force (Fig. 1a). Mental health carers who were not working reported a range of roles, including home duties and retirement, with 7.2% of all mental health carers reporting their main activity as caring. As seen in Fig. 1b and c, more female than male mental health carers worked part-time or were not employed, and a larger proportion reported their main activity as home duties or retirement.

When compared to mental health carers, non-carers had less than half the odds of not being employed (Table 1). However, there were no significant differences in employment rates between mental health carers and other disability carers.

**Table 1:** Simple logistic regression analyses on employment status, hours and occupation for carers and non-carers, by main condition of care recipient

Care recipient disability group	n	% (95% CI)	OR (95% CI)	*p*
*Not employed*				
Mental illness	520	42.3 (36.6–48.1)	1.00	
Physical condition only	1455	38.3 (34.8–42.0)	0.85 (0.64–1.12)	.24
Physical condition with mental illness	577	42.3 (37.6–47.2)	1.00 (0.71–1.41)	.99
Other cognitive/behavioural condition	312	40.0 (32.6–47.9)	0.91 (0.59–1.40)	.66
Not a carer	35,400	24.0 (23.5–24.6)	0.43 (0.34–0.55)	**<.001**
*If employed, works < 16 h per week*				
Mental illness	293	17.2 (12.8–22.8)	1.00	
Physical condition only	887	16.0 (13.6–18.8)	0.92 (0.62–1.37)	.67
Physical condition with mental illness	322	14.6 (10.6–19.8)	0.82 (0.49–1.37)	.45
Other cognitive/behavioural condition	188	13.8 (9.7–19.3)	0.77 (0.47–1.28)	.31
Not a carer	26,699	11.7 (11.3–12.1)	0.63 (0.44–0.91)	**.01**
*If employed, works as a machinery operator, driver or labourer*				
Mental illness	293	22.6 (17.5–28.7)	1.00	
Physical condition only	885^a^	18.4 (15.2–22.0)	0.77 (0.54–1.11)	.16
Physical condition with mental illness	322	23.3 (18.9–28.3)	1.04 (0.68–1.58)	.86
Other cognitive/behavioural condition	188	14.8 (10.1–21.2)	0.59 (0.36–0.97)	**.04**
Not a carer	26,649^a^	15.7 (15.1–16.2)	0.64 (0.46–0.87)	**.006**

OR: odds ratio; CI: confidence interval; *p-*values in **bold** are significant at *p* < .05

^a^ Excludes two carers of physical health only conditions and 50 non-carers who inadequately described their occupation

#### Hours and type of work

Employed mental health carers reported a range of working hours, with 17.2% (95% CI: 12.8–22.8) working 1–15 h per week, 25.5% (95% CI: 19.3–33.0) working 16–34 h, 32.9% (95% CI: 26.4–40.2) 35–40 h and 24.3% (95% CI: 19.7–29.6) 41 or more hours. Compared to employed mental health carers, employed non-carers had significantly lower odds of working fewer than 16 h per week (Table 1). There were no significant differences in working hours between employed carers for different conditions.

Applying high-level occupational groupings, 36.3% (95% CI: 29.5–43.6) of employed mental health carers worked as a manager or professional; 41.1% (95% CI: 35.0–47.6) in a technical, trade, service, sales or clerical role; and 22.6% (95% CI: 17.5–28.7) as a machinery operator, driver or labourer. Employed non-carers and carers for other cognitive/behavioural conditions had significantly lower odds than employed mental health carers of working as a machinery operator, driver or labourer rather than any other technical or professional role (Table 1), but there was no significant difference between mental health and physical health carers.

#### Impact of caring on employment

Within the subgroup of confirmed primary mental health carers (*n* = 137), less than half were employed (43.8, 95% CI: 33.4–54.8). Of primary mental health carers who were not currently employed, only 47.0% (95% CI: 32.7–61.8) worked prior to commencing caring; these rates were similar across primary carers for other conditions (Additional file 1: Table S4). Excluding carers who were not currently employed and did not work prior to caring, more than half of primary mental health carers reported an impact of caring on their working hours: 26.4% (95% CI: 17.2–38.2) stopped working altogether to care, and a further 25.8% (95% CI: 15.6–39.5) had reduced their working hours. This was comparable to other disability groups, for which between 45 and 56% of working primary carers reduced their hours or left employment due to caring (Additional file 1: Table S4). Of employed primary mental health carers, 13.8% (95% CI: 7.0–25.5) left work for at least three months to care for the main person they supported, and 28.9% (95% CI: 17.9–43.2) needed time off work to care.

### Aim 2. Factors related to employment for mental health carers

Table 2 shows the results of the logistic regression models on employment status for mental health carers. Key demographic and caring role characteristics associated with employment status for female mental health carers were their age, education, own disability, and the disability level of the person cared for. Female mental health carers had more than three times lower odds of not being employed if they were aged 35–54 years (compared to 15–34 years) and two to three times higher odds of not being employed if they had: completed secondary education or less, had a disability themselves, or cared for someone with profound or severe limitations in core activities (communication, mobility and self-care). Whether the person they supported received formal services was not significantly associated with employment for female mental health carers.

**Table 2:** Association between demographic and caring role characteristics and not being employed for mental health carers

Carer characteristic	Female carers (*n* = 268)			Male carers (*n* = 238)		
	% not employed (95% CI)	AOR (95% CI)	*p*	% not employed (95% CI)	AOR (95% CI)	*p*
Age group					ns	
15–34 years	64.7 (46.5–79.4)	1.00		36.5 (23.0–52.4)		
35–54 years	40.7 (31.1–51.1)	0.31 (0.11–0.86)	**.03**	24.0 (16.1–34.2)		
55–64 years	65.9 (50.1–79.0)	0.74 (0.24–2.34)	.61	38.2 (26.0–52.0)		
Highest level of education^a^					ns	
Post-secondary degree/certificate	36.6 (27.8–46.4)	1.00		23.4 (16.4–32.4)		
Senior secondary school (Year 11 or 12)	67.9 (53.3–79.7)	3.09 (1.41–6.74)	**.005**	34.9 (21.6–51.0)		
Junior secondary school (Year 10) or less	74.1 (61.9–83.4)	3.86 (1.59–9.39)	**.004**	48.9 (33.8–64.2)		
Carer’s own disability status						
No disability	40.3 (30.6–50.9)	1.00		24.7 (16.6–35.0)	1.00	
Has a disability	68.0 (58.5–76.1)	3.60 (1.68–7.69)	**.001**	48.6 (32.6–64.9)	3.64 (1.07–12.39)	**.04**
Cares for their spouse/partner or adult child		ns				
Cares for another relative/friend only	65.9 (48.9–79.7)			49.3 (34.7–64.0)	1.00	
Cares for their partner/child	49.7 (41.4–57.9)			24.7 (18.7–31.9)	0.38 (0.13–1.12)	.08
Care recipient disability level						
Moderate or less limitation in core activities	42.1 (33.4–51.4)	1.00		16.2 (8.5–28.6)	1.00	
Profound or severe limitation in core activities	64.1 (52.5–74.2)	2.13 (1.02–4.43)	**.04**	44.5 (34.0–55.5)	4.21 (1.45–12.25)	**.009**
Care recipient(s) receipt of any formal services		ns				
Does not receive services	55.3 (42.7–67.3)			44.3 (33.0–56.1)	1.00	
Receives services	51.7 (41.7–61.6)			22.8 (15.9–31.4)	0.34 (0.18–0.65)	**.001**

AOR: adjusted odds ratio; CI: confidence interval; ns: factor was not significantly related to employment at *p* > .10 and was not included in final model

^a^ In Australia, Year 12 is the final year of secondary schooling, generally complete at age 17 or 18

Notes: *p-*values in **bold** are significant at *p* < .05. The following factors were not significantly related to employment status and were removed from the final regression models: (1) for female mental health carers: marital status, rurality, country of birth, primary carer status, number of recipients of care, caring for their partner/child, and care recipient receipt of formal services; (2) for male mental health carers: age group, marital status, rurality, country of birth, education level, primary carer status, and number of recipients of care

For male mental health carers, fewer of the carers’ demographic characteristics were associated with employment status. Rather, male mental health carers had three to four times greater odds of not being employed if they were caring for a person with profound/severe core activity limitations or had a disability themselves, and nearly three times lower odds of not being employed if the person they cared for received any formal services (Table 2). Supplementary regression models controlling for carer disability, relationship to the person supported, and disability level of that person (Additional file 1: Tables S5 and S6) found that male mental health carers had lower odds of not being employed if the person cared for received formal assistance with cognitive or emotional tasks (vs. no assistance with cognitive or emotional tasks; AOR 0.35, 95% CI: 0.19–0.65, *p* = .001), or if that person received any type of formal assistance at least weekly (vs. no formal assistance; AOR 0.23, 95% CI: 0.08–0.68, *p* = .009).

### Aim 3. Unique factors for mental health carers vs. other carers

Initial bivariate comparisons between mental health and other disability carers found that a greater proportion of the former cared for their spouse/partner or adult child, or for a person supported by formal services (Additional file 1: Table S3). More female mental health carers than other female carers had a disability, but fewer were primary carers and a smaller proportion cared for a person with profound/severe activity limitations. A greater proportion of male mental health carers supported two or more people compared to other male carers. These factors were entered into the logistic regression models for all disability carers, along with the factors identified as significantly related to mental health carers’ employment for each gender.

Controlling for these factors, disability group of the person supported (mental illness vs. other) was not significantly associated with employment for male or female carers (Table 3). There were no significant differences between the factors associated with employment for female mental health carers versus for other female carers. For male carers, the characteristics associated with employment were the same for carers of people with mental illness and other conditions. For male carers only, there were insignificant trends towards group differences in the importance of the level of core activity limitation and receipt of formal services by the person cared for, indicating a possible larger effect for mental health carers than for other carers; however, the directions of effects were the same for all carers. A replication of the regression analyses using physical health carers only as the comparison group (excluding other cognitive/behavioural condition carers, who may have similarities to mental health carers) produced the same patterns of significance as the overall analysis.

**Table 3:** Association between demographic and caring role characteristics and not being employed for all disability carers

Carer characteristic	Female carers (*n* = 1485)			Male carers (*n* = 1320)		
	% not employed (95% CI)	AOR (95% CI)	*p*	% not employed (95% CI)	AOR (95% CI)	*p*
Recipient disability group						
Other condition	46.2 (42.7–49.8)	1.00		32.1 (28.8–35.6)	1.00	
Mental illness	53.3 (45.4–61.0)	1.32 (0.91–1.92)	.14	31.3 (25.2–38.2)	1.06 (0.64–1.77)	.81
Age group					n/a	
15–34 years	46.0 (39.7–52.4)	1.00		38.3 (32.7–44.1)		
35–54 years	40.0 (35.6–44.3)	0.65 (0.46–0.93)	**.02**	22.5 (18.7–26.9)		
55–64 years	60.6 (55.8–65.3)	1.27 (0.93–1.74)	.14	37.9 (33.1–42.9)		
Highest level of education					n/a	
Post-secondary degree/certificate	35.1 (31.2–39.2)	1.00		21.1 (18.5–24.0)		
Year 12 or less	61.2 (56.9–65.4)	2.73 (2.11–3.53)	**<.001**	45.9 (41.5–50.4)		
Carer’s own disability status						
No disability	38.9 (35.1–42.8)	1.00		25.2 (22.4–28.2)	1.00	
Has a disability	65.8 (60.6–70.6)	2.93 (2.14–4.00)	**<.001**	50.7 (44.7–56.7)	4.01 (2.83–5.69)	**<.001**
Primary carer status					n/a	
Is not a primary carer	41.0 (37.2–44.9)	1.00		27.9 (24.9–31.1)		
Is a primary carer	56.6 (51.8–61.3)	1.62 (1.17–2.25)	**.005**	45.8 (40.4–51.4)		
Number of recipients of care		n/a				
One	45.9 (42.6–49.2)			30.4 (27.2–33.8)	1.00	
Two or more	52.7 (44.9–60.4)			38.7 (32.0–45.9)	1.50 (0.99–2.28)	.056
Cares for their spouse/partner or adult child		ns				
Cares for another relative/friend only	47.7 (42.8–52.5)			42.6 (37.9–47.4)	1.00	
Cares for their partner/child	47.3 (43.6–50.9)			23.4 (20.4–26.7)	0.34 (0.24–0.47)	**<.001**
Care recipient disability level						
Moderate or less limitation in core activities	38.0 (33.3–42.8)	1.00		23.1 (18.9–27.8)	1.00	
Profound or severe limitation in core activities	52.5 (48.7–56.4)	1.55 (1.14–2.10)	**.006**	38.0 (34.5–41.7)	2.47 (1.43–4.30)	**.002**
Care recipient disability level X recipient disability group interaction		ns				.09
Moderate or less (other condition)	36.7 (30.9–42.9)			24.8 (20.0–30.3)	1.00	
Profound/severe vs. moderate/less (other condition)	50.8 (46.5–55.1)			36.8 (32.7–41.0)	1.54 (1.06–2.24)	**.02**
Moderate or less (mental illness)	42.1 (33.4–51.4)			16.2 (8.5–28.6)	1.00	
Profound/severe vs. moderate/less (mental illness)	64.1 (52.5–74.2)			44.5 (34.0–55.5)	3.97 (1.40–11.29)	**.01**
Care recipient(s) receipt of any formal services						
Does not receive services	48.5 (44.5–52.5)	1.00		34.6 (30.5–38.9)	1.00	
Receives services	46.3 (41.9–50.8)	0.80 (0.62–1.04)	.096	29.5 (26.5–32.7)	0.53 (0.36–0.79)	**.002**
Care recipient(s) receipt of any formal services X recipient disability group interaction		ns				.08
Does not receive services (other condition)	47.3 (43.3–51.5)			33.0 (28.4–37.8)	1.00	
Receives services vs. not (other condition)	45.0 (39.7–50.4)			31.2 (27.6–35.2)	0.77 (0.58–1.03)	.07
Does not receive services (mental illness)	55.3 (42.7–67.3)			44.3 (33.0–56.1)	1.00	
Receives services vs. not (mental illness)	51.7 (41.7–61.6)			22.8 (15.9–31.4)	0.37 (0.17–0.79)	**.01**

AOR: adjusted odds ratio; CI: confidence interval; n/a: not included in the initial model because factor not significantly related to employment in the mental health carer regression model and not significantly different between mental health versus other carers; ns: factor was not significantly related to employment at *p* > .10 and was not included in final model

Notes: *p-*values in **bold** are significant at *p* < .05. The following factors were initially included but were not significantly related to employment status and were therefore removed from the final regression models: (1) for female mental health carers: caring for their partner/child, and all interactions between recipient disability group and covariates; (2) for male mental health carers: interaction terms between recipient disability group and all covariates except care recipient disability level and receipt of formal services

## Discussion

In Australia in 2015, working-age co-resident carers of adults with mental illness were significantly less likely to be employed and were employed for fewer hours and in lower-level occupations than adults without caring responsibilities. This is consistent with previous research demonstrating lower employment rates for informal carers compared to non-carers [1], and earlier research on Australian mental health carers suggesting low employment rates for this group [9, 11].

The main characteristics associated with employment for female mental health carers were age, education level, disability, and disability level of the person cared for. For male mental health carers, having a disability, disability level of the person cared for, and the latter’s receipt of formal assistance were associated with employment. These results are similar to previous research on carers internationally [1, 6, 30], although other factors from previous studies, such as being the primary carer and the number of people supported, were not the most important for this 2015 SDAC carer group. For men, significant factors were associated with the person cared for and caring role, whereas for female carers their own socio-demographic characteristics were more prominent. A greater proportion of female than male mental health carers were not in the labour force due to home duties and child care, retirement or being permanently unable to work. The stronger association between their own characteristics and employment for female carers likely reflects this greater diversity of other roles and their influence on workforce participation.

Male mental health carers were more likely to be employed if the person they supported received assistance from organised services, and this was true specifically of assistance with cognitive or emotional tasks, and services provided at least weekly. Further, both male and female mental health carers were less likely to be employed if the person they cared for had a higher level of disability. These results support and extend previous UK research on all carers, including longitudinal follow-up, which found that a range of services provided to people with disabilities were associated with their carers’ employment [6, 18]. Improving the availability and impact of psychosocial support services for people with mental illness may therefore assist their carers to maintain employment, but possibly more so for male than female carers.

Contrary to our expectation that the focus of mental health caring on emotional assistance and episodic support would interfere more with carers’ employment, there were no significant differences in employment rates between mental health carers and carers for people with other cognitive/behavioural conditions or physical conditions with or without secondary mental illness. The analysis did not show any factors related to employment that were unique to mental health carers. The employment picture was remarkably similar across carers for all disabilities, suggesting that the degree of impairment of the person cared for, available supports, and the carer’s own personal circumstances are more influential for employment status, perhaps through their impact on direct time commitments, than the nature of caring tasks or subjective burden of caring, which might be more relevant for subjective distress experienced in balancing employment and caring. Thus this study focused on a cross-sectional analysis of current employment status did not provide evidence that mental health carers need specially targeted programs to support them to work, separate from those for other carers. It is possible that while physical and mental health carers may provide different supports and experience caring differently, these roles and stressors have a similar impact on maintaining employment. It is also possible that caring role differences may affect other aspects of carers’ employment not measured in this study, which did not include carers’ subjective experience of their employment, caring or available supports. Previous research has found that stress associated with mental health caring contributes to poorer work performance [11], and that schizophrenia carers report higher absenteeism, presenteeism and burden compared to other carers [17]. Future research could explore potential differences in these other employment-related factors among carers for different conditions in Australia, or the mediating effects of different caring tasks or crisis-related care on subjective and objective experiences of employment.

The study results seem timely given the current roll-out of the National Disability Insurance Scheme (NDIS) in Australia, which is moving disability support services from grant-funded programs accessed via non-government organisations to individual packages of care based on needs assessments. The NDIS is required to take into account both what support is reasonable to expect families and carers to provide, as well as risks to the wellbeing of the person with disability and their carer from continuing pre-existing intensive caring arrangements [41]. However, widespread concern about the appropriateness of the Scheme for individuals with psychosocial disabilities and their carers has prompted a review of processes [42, 43]. Given the clear benefits to mental health carers and society from their participation in employment [23, 24, 27], it is critical that the implementation of the NDIS maintains or improves the level of support available for carers and people with psychosocial disabilities, to prevent carers from feeling they have no choice but to leave employment in order to support their loved ones. Further, consideration is needed of appropriate support arrangements for people with mental illness and their carers who are not eligible for the NDIS. Better access to community support services for people with mental illness will never completely substitute for informal caring, but would help to take the pressure off carers and allow them to better manage their multiple roles [1, 6, 44].

### Limitations

The cross-sectional nature of the 2015 SDAC means that all analyses in this study were correlational and did not distinguish the direction of impact between carer and caring role characteristics and carers’ employment. Carers may self-select to caring based in part on lower opportunity costs, being more likely to choose caring over employment if they are already near retirement age, in a less rewarding job or in poor health [1]. However, a number of longitudinal studies have shown that this gap widens over time, with caring having a negative impact on later employment [6, 30, 45–47]. Unfortunately, available Australian longitudinal studies which include carers do not record the condition of the person cared for, meaning the 2015 SDAC currently provides the most up-to-date, comprehensive and nationally representative data on Australian mental health carers. This analysis should be considered an initial exploration of available data which could be replicated in the future when longitudinal studies of mental health carers are available. It is recommended that questions about the condition of the person being cared for be added to recurrent surveys such as the Household, Income and Labour Dynamics in Australia (HILDA) Survey or the Australian Longitudinal Study on Women’s Health to allow such analyses.

The analysis was restricted to co-resident carers of adults with mental illness. In the 2015 SDAC dataset, the conditions of the person supported were only available for carers living with that person. This is likely to have produced a stronger relationship between caring role characteristics and employment than might be expected in a broader sample of carers, since the impact of caring on employment has been found to be greater for co-resident carers [1, 6, 34]. Mental health carers who do not live with their care recipient may still face significant challenges; for some carers these could cause more conflict with employment due to additional time needed to travel to the person they support. The 2015 SDAC identified carers and their basic information via household informants and only primary carers were personally interviewed. This method may have missed or misidentified carers because carers, their families and the people they support can be reluctant or unable to recognise and label their caring role [48]. The sample was also restricted to carers of adults aged 15 and over. The relationship between caring and employment for parents with dependent children is likely to be complicated by the demands of normal parenting, and with the available cross-sectional data it was not possible to distinguish the impact of these different needs.

Certain factors which may be related to carers’ employment status were not available for analysis, including experience of stigma, history of employment, and caring hours. Hours of care were only recorded for co-resident primary carers in the 2015 SDAC, so other indicators of caring intensity were included in the regression analyses for the broader group of primary and secondary carers (such as caring for a close relative, the number of people supported, and the care recipient’s level of impairment).

## Conclusions

Co-resident mental health carers had significantly lower employment rates than non-carers in 2015 which, alongside earlier studies, suggests a continuing disadvantage associated with caring. Australian initiatives have attempted to support carers of children and people with disabilities in the workforce through encouraging employers to provide flexible work arrangements, and through funding limited support services for carers such as the Department of Social Services’ Carers and Work program [49, 50]. Carers generally report needing better access to services for the person they support to help manage their own employment and overall caring burden [3, 5, 6], and mental health carers in particular have reported inadequate service assistance and higher unmet needs than their other carer counterparts [9, 10]. The results of this study highlight the need to consider the carer’s employment journey in the context of their caring role, particularly the disability level of and supports received by the person cared for.

## Additional file

Additional file 1: **Table S1.** Classification of main disabling condition of recipient of care from the 2015 Survey of Disability, Ageing and Carers. **Table S2.** Relationships between pairs of independent and dependent variables for multivariate logistic regression analyses, by carer sex. **Table S3.** Characteristics of co-resident carers aged 15–64 years, by main condition of the adult being cared for. **Table S4.** Impact of caring on employment for co-resident primary carers aged 15–64 years, by main condition of the adult being cared for. **Table S5.** Supplementary logistic regression analyses of association between recipient types of formal assistance, unmet need for assistance, other carer characteristics and not being employed for co-resident carers aged 15–64 years of adults with mental illness. **Table S6.** Supplementary logistic regression analyses of association between recipient frequency of formal assistance, unmet need for assistance, other carer characteristics and not being employed for co-resident carers aged 15–64 years of adults with mental illness. (DOCX 102 kb)

## Abbreviations

ABS: Australian Bureau of Statistics
AOR: Adjusted odds ratio
CI: Confidence interval
HILDA: Household, Income and Labour Dynamics in Australia Survey
SDAC: Survey of Disability, Ageing and Carers
