# Supplementary material for: Employment disadvantage and associated factors for informal carers of adults with mental illness: are they like other disability carers?
Source: BMC Public Health. 2019 May 16;19:587. doi: 10.1186/s12889-019-6822-1 (PMC6524279; doi:10.1186/s12889-019-6822-1)
Supplement: Supplementary file 1 — Table S1. Classification of main disabling condition of recipient of care from the 2015 Survey of Disability, Ageing and Carers. Table S2. Relationships between pairs of independent and dependent variables for multivariate logistic regression analyses, by carer sex. Table S3. Characteristics of co-resident carers aged 15–64 years, by main condition of the adult being cared for. Table S4. Impact of caring on employment for co-resident primary carers aged 15–64 years, by main condition of the adult being cared for. Table S5. Supplementary logistic regression analyses of association between recipient types of formal assistance, unmet need for assistance, other carer characteristics and not being employed for co-resident carers aged 15–64 years of adults with mental illness. Table S6. Supplementary logistic regression analyses of association between recipient frequency of formal assistance, unmet need for assistance, other carer characteristics and not being employed for co-resident carers aged 15–64 years of adults with mental illness. (DOCX 102 kb) [file 12889_2019_6822_MOESM1_ESM.docx]

**Additional file 1**

**Table S1.** Classification of main disabling condition of recipient of care from the 2015 Survey of Disability, Ageing and Carers

| 1. Mental illness |
| --- |
| 0500. Mental and behavioural disorders n.f.d.  0512. Schizophrenia  0513. Depression/mood affective disorders (excluding postnatal depression)  0519. Other psychoses  0521. Phobic and anxiety disorders  0522. Nervous tension/stress  0529. Other neurotic, stress related and somatoform disorders  0591. Mental disorders due to alcohol and other psychoactive substance use  0594. Adult personality and behavioural disorders  0595. Attention deficit disorder/hyperactivity  0599. Other mental and behavioural disorders |
| 2. Other cognitive/behavioural condition |
| 0511. Dementia  0530. Intellectual and developmental disorders n.e.c.  0531. Mental retardation/intellectual disability  0532. Autism and related disorders (including Rett's syndrome and Asperger's syndrome)  0539. Other developmental/learning disorders  0596. Speech impediment  0605. Alzheimer's disease  1605. Congenital brain damage/malformation  1709. Memory loss n.f.d.  1711. Insomnia n.f.d.  1798. Agitation or confusion n.f.d.  1801. Head injury/acquired brain damage  1908. Memory problems or periods of confusion |
| 3. Physical condition |
| 0199. Certain infectious and parasitic diseases  0203. Skin cancer  0204. Breast cancer  0205. Prostate cancer  0211. Bowel/colorectal cancer  0299. Other neoplasms (including benign tumours)  0301. Anaemia  0399. Other diseases of the blood and blood forming organs and certain disorders involving the immune system  0401. Disorders of the thyroid gland  0402. Diabetes  0403. Obesity  0404. High cholesterol  0499. Other endocrine, nutritional and metabolic disorders  0602. Systemic atrophies primarily affecting the central nervous system  0604. Parkinson's disease  0606. Brain disease/disorders—acquired  0607. Multiple sclerosis  0608. Epilepsy  0609. Migraine  0611. Cerebral palsy  0612. Paralysis  0699. Other diseases of the nervous system (including T.I.A.’s)  0702. Cataracts  0703. Retinal disorders/defects  0704. Glaucoma  0707. Sight loss  0708. Macular degeneration  0799. Other diseases of the eye and adnexa  0802. Diseases of the middle ear and mastoid  0803. Diseases of the inner ear (except noise induced deafness)  0804. Tinnitus  0810. Deafness/hearing loss  0811. Deafness/hearing loss—noise induced  0812. Deafness/hearing loss—congenital  0813. Deafness/hearing loss—due to accident  0819. Other deafness/hearing loss  0899. Other diseases of the ear and mastoid process  0910. Heart disease  0913. Angina  0914. Myocardial infarction (heart attack)  0919. Other heart diseases  0920. Diseases of the circulatory system n.e.c.  0922. Hypertension (high blood pressure)  0923. Stroke  0925. Hypotension (low blood pressure)  0929. Other diseases of the circulatory system  1004. Emphysema  1005. Asthma  1007. Chronic Airflow Limitation (CAL)  1099. Other diseases of the respiratory system  1101. Stomach/duodenal ulcer  1102. Abdominal hernia (except congenital)  1103. Enteritis and colitis  1104. Other diseases of the intestine  1106. Diseases of the liver  1199. Diseases of the digestive system  1201. Skin and subcutaneous tissue infections  1202. Skin allergies (Dermatitis and Eczema)  1299. Other diseases of the skin and subcutaneous tissue  1301. Arthritis and related disorders  1303. Back problems (dorsopathies)  1304. Repetitive strain injury/occupational overuse syndrome  1306. Other soft tissue/muscle disorders (including Rheumatism)  1307. Osteoporosis  1399. Other disorders of the musculoskeletal system and connective tissue  1401. Kidney and urinary system (bladder) disorders (except incontinence)  1402. Stress/urinary incontinence  1403. Prostate disorders  1405. Menopause disorders  1499. Other diseases of the genitourinary system  1599. Certain conditions originating in the perinatal period  1699. Other congenital malformations and deformations  1701. Breathing difficulties/shortness of breath  1704. Pain n.f.d.  1705. Unspecified speech difficulties  1708. Blackouts, fainting, convulsions n.e.c.  1710. Incontinence n.f.d.  1713. Dysphagia (difficulty in swallowing)  1799. Other symptoms and signs n.e.c.  1802. Arm/hand/shoulder damage from injury/accident  1804. Leg/knee/foot/hip damage from injury/accident  1805. Amputation of toe/foot/leg  1808. Complications/consequences of surgery and medical care n.e.c.  1899. Other injury, poisoning and consequences of external causes  1901. Limited use of arms or fingers  1902. Difficulty gripping or holding things  1903. Limited use of feet or legs  1904. Restriction in physical activity or physical work  1905. Has disfigurement or deformity  1906. Receiving treatment/medication for other long term condition  1907. Has other long term condition |

n.f.d – not further defined

n.e.c. – not elsewhere classified

**Table S2.** Relationships between pairs of independent and dependent variables for multivariate logistic regression analyses, by carer sex (moderate associations >.30 in **bold**)

| **Variable 1** | **Variable 2** | **Cramer’s *V*** | ***df*** | ***p*** |
| --- | --- | --- | --- | --- |
| **FEMALE MENTAL HEALTH CARERS (n=268)** | | | | |
| Employment status | Age group | .30 | 2 | <.001 |
|  | Marital status | -.04 | 1 | .50 |
|  | Rurality | -.06 | 1 | .36 |
|  | Country of birth | .13 | 1 | .03 |
|  | **Education level** | **.34** | **2** | **<.001** |
|  | Carer disability | .30 | 1 | <.001 |
|  | Primary carer | .08 | 1 | .18 |
|  | Number of recipients | .01 | 1 | .83 |
|  | Cares for partner/child | -.10 | 1 | .11 |
|  | Recipient disability level | .15 | 1 | .01 |
|  | Recipient formal services | -.03 | 1 | .60 |
| Age group | **Marital status** | **.37** | **2** | **<.001** |
|  | Rurality | .06 | 2 | .66 |
|  | Country of birth | .18 | 2 | .01 |
|  | Education level^1^ | .17 | 4 | .003 |
|  | Carer disability | .09 | 2 | .38 |
|  | Primary carer | .11 | 2 | .19 |
|  | Number of recipients | .06 | 2 | .57 |
|  | **Cares for partner/child** | **.52** | **2** | **<.001** |
|  | Recipient disability level | .03 | 2 | .86 |
|  | Recipient formal services | .04 | 2 | .76 |
| Marital status | Rurality | -.02 | 1 | .79 |
|  | Country of birth | .13 | 1 | .03 |
|  | Education level | .13 | 2 | .11 |
|  | Carer disability | -.10 | 1 | .11 |
|  | Primary carer | -.03 | 1 | .59 |
|  | Number of recipients | .11 | 1 | .07 |
|  | **Cares for partner/child** | **.39** | **1** | **<.001** |
|  | Recipient disability level | -.08 | 1 | .18 |
|  | Recipient formal services | -.06 | 1 | .36 |
| Rurality | Country of birth | -.20 | 1 | .001 |
|  | Education level | .05 | 2 | .67 |
|  | Carer disability | -.10 | 1 | .11 |
|  | Primary carer | .08 | 1 | .17 |
|  | Number of recipients | -.01 | 1 | .88 |
|  | Cares for partner/child | -.01 | 1 | .89 |
|  | Recipient disability level | -.02 | 1 | .71 |
|  | Recipient formal services | -.09 | 1 | .14 |
| Country of birth | Education level | .03 | 2 | .87 |
|  | Carer disability | .05 | 1 | .46 |
|  | Primary carer | .04 | 1 | .47 |
|  | Number of recipients | .01 | 1 | .85 |
|  | Cares for partner/child | .03 | 1 | .57 |
|  | Recipient disability level | -.02 | 1 | .67 |
|  | Recipient formal services | -.06 | 1 | .32 |
| Education level | Carer disability | .13 | 2 | .11 |
|  | Primary carer | .15 | 2 | .05 |
|  | Number of recipients | .06 | 2 | .57 |
|  | Cares for partner/child | .12 | 2 | .14 |
|  | Recipient disability level | .20 | 2 | .01 |
|  | Recipient formal services | .08 | 2 | .43 |
| Carer disability | Primary carer | .02 | 1 | .71 |
|  | Number of recipients | .09 | 1 | .13 |
|  | Cares for partner/child | .06 | 1 | .32 |
|  | Recipient disability level | .05 | 1 | .39 |
|  | Recipient formal services | -.01 | 1 | .81 |
| Primary carer | Number of recipients | .08 | 1 | .19 |
|  | Cares for partner/child | -.06 | 1 | .29 |
|  | **Recipient disability level** | **.57** | **1** | **<.001** |
|  | Recipient formal services | .03 | 1 | .64 |
| Number of recipients | Cares for partner/child | .05 | 1 | .37 |
|  | Recipient disability level | .13 | 1 | .03 |
|  | Recipient formal services | .04 | 1 | .54 |
| Cares for partner/child | Recipient disability level | -.16 | 1 | .01 |
|  | Recipient formal services | -.05 | 1 | .40 |
| Recipient disability level | Recipient formal services | .05 | 1 | .39 |
| **MALE MENTAL HEALTH CARERS (n=238)** | | | | |
| Employment status | Age group | .14 | 2 | .10 |
|  | Marital status | -.20 | 1 | .003 |
|  | Rurality | .01 | 1 | .92 |
|  | Country of birth | .07 | 1 | .31 |
|  | Education level | .22 | 2 | .003 |
|  | Carer disability | .25 | 1 | <.001 |
|  | Primary carer | .14 | 1 | .03 |
|  | Number of recipients | .06 | 1 | .33 |
|  | Cares for partner/child | -.27 | 1 | <.001 |
|  | Recipient disability level | .25 | 1 | <.001 |
|  | Recipient formal services | -.19 | 1 | .003 |
| Age group | **Marital status** | **.58** | **2** | **<.001** |
|  | Rurality | .12 | 2 | .16 |
|  | Country of birth | .07 | 2 | .57 |
|  | Education level^1^ | .27 | 4 | <.001 |
|  | Carer disability | .13 | 2 | .14 |
|  | Primary carer | .20 | 2 | .01 |
|  | Number of recipients | .05 | 2 | .74 |
|  | **Cares for partner/child** | **.64** | **2** | **<.001** |
|  | Recipient disability level | .11 | 2 | .23 |
|  | Recipient formal services | .03 | 2 | .90 |
| Marital status | Rurality | .07 | 1 | .29 |
|  | Country of birth | .09 | 1 | .19 |
|  | Education level | .22 | 2 | .003 |
|  | Carer disability | -.09 | 1 | .15 |
|  | Primary carer | .08 | 1 | .19 |
|  | Number of recipients | .11 | 1 | .08 |
|  | **Cares for partner/child** | **.58** | **1** | **<.001** |
|  | Recipient disability level | .01 | 1 | .83 |
|  | Recipient formal services | -.02 | 1 | .80 |
| Rurality | Country of birth | -.22 | 1 | .001 |
|  | Education level | .17 | 2 | .03 |
|  | Carer disability | .06 | 1 | .35 |
|  | Primary carer | -.04 | 1 | .49 |
|  | Number of recipients | -.12 | 1 | .06 |
|  | Cares for partner/child | .05 | 1 | .47 |
|  | Recipient disability level | .02 | 1 | .74 |
|  | Recipient formal services | -.20 | 1 | .003 |
| Country of birth | Education level | .08 | 2 | .50 |
|  | Carer disability | -.09 | 1 | .15 |
|  | Primary carer | .06 | 1 | .35 |
|  | Number of recipients | -.004 | 1 | .96 |
|  | Cares for partner/child | .03 | 1 | .70 |
|  | Recipient disability level | .08 | 1 | .24 |
|  | Recipient formal services | -.06 | 1 | .37 |
| Education level | Carer disability | .08 | 2 | .45 |
|  | Primary carer | .06 | 2 | .65 |
|  | Number of recipients | .13 | 2 | .12 |
|  | **Cares for partner/child** | **.30** | **2** | **<.001** |
|  | Recipient disability level | .08 | 2 | .43 |
|  | Recipient formal services | .16 | 2 | .04 |
| Carer disability | Primary carer | .13 | 1 | .05 |
|  | Number of recipients | .11 | 1 | .08 |
|  | Cares for partner/child | .02 | 1 | .79 |
|  | Recipient disability level | .03 | 1 | .61 |
|  | Recipient formal services | .05 | 1 | .43 |
| Primary carer | Number of recipients | .09 | 1 | .15 |
|  | Cares for partner/child | .11 | 1 | .08 |
|  | **Recipient disability level** | **.45** | **1** | **<.001** |
|  | Recipient formal services | -.06 | 1 | .37 |
| Number of recipients | Cares for partner/child | .06 | 1 | .38 |
|  | Recipient disability level | .22 | 1 | .001 |
|  | Recipient formal services | .08 | 1 | .20 |
| Cares for partner/child | Recipient disability level | -.14 | 1 | .03 |
|  | Recipient formal services | .07 | 1 | .25 |
| Recipient disability level | Recipient formal services | -.09 | 1 | .16 |
| **FEMALE CARERS (all disability groups; n=1,485)** | | | | |
| Employment status | Recipient disability group | .04 | 1 | .10 |
|  | Age group | .20 | 2 | <.001 |
|  | Marital status | -.03 | 1 | .26 |
|  | Rurality | .04 | 1 | .13 |
|  | Country of birth | .07 | 1 | .004 |
|  | Education level | .27 | 1 | <.001 |
|  | Carer disability | .26 | 1 | <.001 |
|  | Primary carer | .15 | 1 | <.001 |
|  | Number of recipients | .04 | 1 | .16 |
|  | Cares for partner/child | -.01 | 1 | .64 |
|  | Recipient disability level | .14 | 1 | <.001 |
|  | Recipient formal services | .001 | 1 | .98 |
| Recipient disability group | Age group | .06 | 2 | .08 |
|  | Marital status | -.03 | 1 | .33 |
|  | Rurality | .03 | 1 | .27 |
|  | Country of birth | -.02 | 1 | .52 |
|  | Education level | -.01 | 1 | .75 |
|  | Carer disability | .13 | 1 | <.001 |
|  | Primary carer | -.07 | 1 | .008 |
|  | Number of recipients | .06 | 1 | .02 |
|  | Cares for partner/child | .15 | 1 | <.001 |
|  | Recipient disability level | -.13 | 1 | <.001 |
|  | Recipient formal services | .09 | 1 | <.001 |
| Age group | **Marital status** | **.42** | **2** | **<.001** |
|  | Rurality | .07 | 2 | .04 |
|  | Country of birth | .10 | 2 | .001 |
|  | Education level | .16 | 2 | <.001 |
|  | Carer disability | .14 | 2 | <.001 |
|  | Primary carer | .23 | 2 | <.001 |
|  | Number of recipients | .06 | 2 | .05 |
|  | **Cares for partner/child** | **.52** | **2** | **<.001** |
|  | Recipient disability level | .06 | 2 | .05 |
|  | Recipient formal services | .03 | 2 | .44 |
| Marital status | Rurality | .02 | 1 | .36 |
|  | Country of birth | .16 | 1 | <.001 |
|  | Education level | -.05 | 1 | .05 |
|  | Carer disability | -.05 | 1 | .04 |
|  | Primary carer | .11 | 1 | <.001 |
|  | Number of recipients | .03 | 1 | .30 |
|  | **Cares for partner/child** | **.52** | **1** | **<.001** |
|  | Recipient disability level | .03 | 1 | .21 |
|  | Recipient formal services | -.03 | 1 | .21 |
| Rurality | Country of birth | -.21 | 1 | <.001 |
|  | Education level | .04 | 1 | .09 |
|  | Carer disability | .07 | 1 | .01 |
|  | Primary carer | .04 | 1 | .09 |
|  | Number of recipients | .01 | 1 | .82 |
|  | Cares for partner/child | .13 | 1 | <.001 |
|  | Recipient disability level | -.04 | 1 | .18 |
|  | Recipient formal services | -.10 | 1 | <.001 |
| Country of birth | Education level | -.05 | 1 | .046 |
|  | Carer disability | -.08 | 1 | .003 |
|  | Primary carer | .02 | 1 | .45 |
|  | Number of recipients | -.06 | 1 | .02 |
|  | Cares for partner/child | -.01 | 1 | .75 |
|  | Recipient disability level | -.004 | 1 | .88 |
|  | Recipient formal services | -.06 | 1 | .03 |
| Education level | Carer disability | .09 | 1 | .001 |
|  | Primary carer | .03 | 1 | .31 |
|  | Number of recipients | -.05 | 1 | .07 |
|  | Cares for partner/child | -.03 | 1 | .23 |
|  | Recipient disability level | .05 | 1 | .04 |
|  | Recipient formal services | -.05 | 1 | .04 |
| Carer disability | Primary carer | .05 | 1 | .04 |
|  | Number of recipients | .08 | 1 | .001 |
|  | Cares for partner/child | .13 | 1 | <.001 |
|  | Recipient disability level | .02 | 1 | .54 |
|  | Recipient formal services | .04 | 1 | .15 |
| Primary carer | Number of recipients | -.01 | 1 | .57 |
|  | Cares for partner/child | .08 | 1 | .001 |
|  | **Recipient disability level** | **.53** | **1** | **<.001** |
|  | Recipient formal services | .11 | 1 | <.001 |
| Number of recipients | Cares for partner/child | .11 | 1 | <.001 |
|  | Recipient disability level | .09 | 1 | .001 |
|  | Recipient formal services | .15 | 1 | <.001 |
| Cares for partner/child | Recipient disability level | -.08 | 1 | .003 |
|  | Recipient formal services | -.04 | 1 | .11 |
| Recipient disability level | Recipient formal services | .15 | 1 | <.001 |
| **MALE CARERS (all disability groups; n=1,320)** | | | | |
| Employment status | Recipient disability group | .01 | 1 | .64 |
|  | Age group | .17 | 2 | <.001 |
|  | Marital status | -.19 | 1 | <.001 |
|  | Rurality | .04 | 1 | .13 |
|  | Country of birth | .04 | 1 | .18 |
|  | Education level | .26 | 1 | <.001 |
|  | Carer disability | .27 | 1 | <.001 |
|  | Primary carer | .16 | 1 | <.001 |
|  | Number of recipients | .04 | 1 | .12 |
|  | Cares for partner/child | -.21 | 1 | <.001 |
|  | Recipient disability level | .15 | 1 | <.001 |
|  | Recipient formal services | -.04 | 1 | .19 |
| Recipient disability group | Age group | .05 | 2 | .16 |
|  | Marital status | .05 | 1 | .09 |
|  | Rurality | .04 | 1 | .12 |
|  | Country of birth | .01 | 1 | .80 |
|  | Education level | -.01 | 1 | .73 |
|  | Carer disability | .04 | 1 | .20 |
|  | Primary carer | -.01 | 1 | .71 |
|  | Number of recipients | .13 | 1 | <.001 |
|  | Cares for partner/child | .16 | 1 | <.001 |
|  | Recipient disability level | -.03 | 1 | .20 |
|  | Recipient formal services | .11 | 1 | <.001 |
| Age group | **Marital status** | **.50** | **2** | **<.001** |
|  | Rurality | .07 | 2 | .03 |
|  | Country of birth | .10 | 2 | .001 |
|  | Education level | .18 | 2 | <.001 |
|  | Carer disability | .21 | 2 | <.001 |
|  | Primary carer | .17 | 2 | <.001 |
|  | Number of recipients | .04 | 2 | .39 |
|  | **Cares for partner/child** | **.51** | **2** | **<.001** |
|  | Recipient disability level | .02 | 2 | .69 |
|  | Recipient formal services | .05 | 2 | .20 |
| Marital status | Rurality | .01 | 1 | .76 |
|  | Country of birth | .15 | 1 | <.001 |
|  | Education level | -.26 | 1 | <.001 |
|  | Carer disability | .06 | 1 | .03 |
|  | Primary carer | .01 | 1 | .60 |
|  | Number of recipients | .06 | 1 | .03 |
|  | **Cares for partner/child** | **.63** | **1** | **<.001** |
|  | Recipient disability level | -.03 | 1 | .23 |
|  | Recipient formal services | .03 | 1 | .25 |
| Rurality | Country of birth | -.26 | 1 | <.001 |
|  | Education level | .04 | 1 | .19 |
|  | Carer disability | .11 | 1 | <.001 |
|  | Primary carer | -.02 | 1 | .57 |
|  | Number of recipients | -.02 | 1 | .48 |
|  | Cares for partner/child | .10 | 1 | <.001 |
|  | Recipient disability level | -.01 | 1 | .65 |
|  | Recipient formal services | -.07 | 1 | .01 |
| Country of birth | Education level | -.05 | 1 | .06 |
|  | Carer disability | -.06 | 1 | .02 |
|  | Primary carer | .03 | 1 | .36 |
|  | Number of recipients | -.05 | 1 | .07 |
|  | Cares for partner/child | .03 | 1 | .27 |
|  | Recipient disability level | .04 | 1 | .16 |
|  | Recipient formal services | -.02 | 1 | .47 |
| Education level | Carer disability | .07 | 1 | .008 |
|  | Primary carer | -.02 | 1 | .46 |
|  | Number of recipients | -.02 | 1 | .46 |
|  | Cares for partner/child | -.25 | 1 | <.001 |
|  | Recipient disability level | .03 | 1 | .32 |
|  | Recipient formal services | -.02 | 1 | .54 |
| Carer disability | Primary carer | .08 | 1 | .002 |
|  | Number of recipients | .04 | 1 | .16 |
|  | Cares for partner/child | .12 | 1 | <.001 |
|  | Recipient disability level | .03 | 1 | .34 |
|  | Recipient formal services | .02 | 1 | .43 |
| Primary carer | Number of recipients | .03 | 1 | .36 |
|  | Cares for partner/child | .05 | 1 | .10 |
|  | **Recipient disability level** | **.42** | **1** | **<.001** |
|  | Recipient formal services | .08 | 1 | .004 |
| Number of recipients | Cares for partner/child | .07 | 1 | .01 |
|  | Recipient disability level | .14 | 1 | <.001 |
|  | Recipient formal services | .15 | 1 | <.001 |
| Cares for partner/child | Recipient disability level | -.17 | 1 | <.001 |
|  | Recipient formal services | .003 | 1 | .92 |
| Recipient disability level | Recipient formal services | .14 | 1 | <.001 |

^1^ Although age group and education level are both ordinal, there was a non-monotonic relationship between the two variables and so a test of ranked association was deemed inappropriate.

**Table S3.** Characteristics of co-resident carers aged 15-64 years, by main condition of the adult being cared for**^a^**

| **Carer characteristic** | **Female co-resident carer % (95% CI)** | | |  | **Male co-resident carer % (95% CI)** | | |
| --- | --- | --- | --- | --- | --- | --- | --- |
|  | **Mental illness (n=268)** | **Other condition (n=1,217)** | **χ^2^ (df), *p*** |  | **Mental illness (n=238)** | **Other condition (n=1,082)** | **χ^2^ (df), *p*** |
| Age group |  |  | 204.50 (2), .19 |  |  |  | 202.19 (2), .36 |
| 15-34 years | 20.7 (15.7-26.8) | 26.8 (24.1-29.6) |  |  | 31.4 (24.8-38.8) | 33.5 (30.0-37.2) |  |
| 35-54 years | 49.2 (42.3-56.1) | 44.8 (41.7-48.0) |  |  | 44.6 (37.5-52.0) | 38.1 (34.7-41.7) |  |
| 55-64 years | 30.1 (23.8-37.3) | 28.4 (25.9-31.0) |  |  | 24.0 (17.1-32.6) | 28.3 (25.3-31.6) |  |
| Married | 52.5 (45.6-59.3) | 55.4 (52.1-58.7) | 36.60 (1), .47 |  | 56.5 (49.2-63.5) | 49.7 (46.2-53.2) | 199.10 (1), .11 |
| Lives in inner regional/other area (not major city) | 36.7 (29.8-44.2) | 31.5 (28.4-34.8) | 128.58 (1), .22 |  | 31.0 (23.4-39.7) | 30.1 (27.0-33.3) | 4.32 (1), .84 |
| Born outside Australia | 22.7 (17.3-29.2) | 25.9 (23.3-28.8) | 57.85 (1), .33 |  | 28.0 (21.2-36.1) | 25.8 (23.2-28.7) | 26.47 (1), .57 |
| Highest level of education: Secondary school (Year 12) or less | 47.4 (40.3-54.5) | 47.1 (43.8-50.4) | 0.34 (1), .94 |  | 43.6 (34.8-52.7) | 43.8 (41.0-46.7) | 0.27 (1), .96 |
| Carer has a disability | 46.8 (40.2-53.5) | 28.6 (25.8-31.6) | 1611.33 (1), **<.001** |  | 27.8 (21.9-34.7) | 26.4 (23.5-29.6) | 11.40 (1), .70 |
| Is a confirmed primary carer | 32.2 (25.6-39.8) | 42.7 (39.4-46.1) | 473.92 (1), **.01** |  | 23.4 (18.1-29.7) | 22.7 (20.2-25.5) | 2.68 (1), .85 |
| More than one care recipient | 26.4 (20.9-32.8) | 21.2 (18.7-23.8) | 170.55 (1), .09 |  | 29.6 (22.6-37.8) | 16.7 (14.3-19.4) | 1175.53 (1), **<.001** |
| Cares for their partner/adult child | 77.9 (71.6-83.1) | 60.5 (57.5-63.4) | 1369.81 (1), **<.001** |  | 73.1 (66.1-79.1) | 51.7 (48.4-55.0) | 1990.68 (1), **<.001** |
| Recipient has profound or severe limitation in core activities | 50.8 (42.8-58.7) | 67.8 (64.7-70.7) | 1340.59 (1), **<.001** |  | 53.5 (44.5-62.2) | 61.0 (57.5-64.4) | 251.39 (1), .11 |
| Care recipient(s) receives any formal services | 56.8 (48.8-64.5) | 47.2 (43.2-51.3) | 387.71 (1), **.03** |  | 60.1 (51.6-68.0) | 49.7 (45.2-54.1) | 465.91 (1), **.02** |

^a^ Excludes 59 carers whose education level was ‘not determined’.

**Table S4.** Impact of caring on employment for co-resident primary carers aged 15-64 years, by main condition of the adult being cared for

|  | **Primary carer % (95% CI)** | | | |
| --- | --- | --- | --- | --- |
|  | **Mental illness  (n=137)** | **Other cognitive/ behavioural condition (n=119)** | **Physical health with secondary mental illness (n=232)** | **Physical health only (n=470)** |
| Employed | 43.8 (33.4-54.8) | 45.5 (33.9-57.6) | 44.5 (37.6-51.7) | 49.4 (44.4-54.4) |
| Worked prior to caring (if not employed) | 47.0 (32.7-61.8) | 53.6 (39.4-67.3) | 53.8 (45.7-61.8) | 46.9 (40.6-53.3) |
| Impact of caring on working hours (if employed or worked prior to caring) |  |  |  |  |
| No reduction in hours^1^ | 47.8 (34.1-61.9) | 44.1 (33.6-55.2) | 51.1 (43.6-58.6) | 55.4 (48.9-61.8) |
| Reduced working hours | 25.8 (15.6-39.5) | 31.5 (21.5-43.7) | 21.0 (15.3-28.2) | 23.6 (18.5-29.7) |
| Stopped working to care | 26.4 (17.2-38.2) | 24.3 (15.0-37.0) | 27.8 (21.0-35.8) | 21.0 (17.0-25.5) |
| Has had to leave work for 3+ months to care (if employed) | 13.8 (7.0-25.5) | 21.3 (10.0-39.6) | 13.3 (7.4-22.8) | 10.9 (6.9-16.9) |
| Needs time off work to care (if employed) | 28.9 (17.9-43.2) | 33.6 (20.5-49.9) | 35.8 (27.3-45.3) | 38.5 (31.8-45.7) |

^1^ Includes primary carers who have maintained or in some cases increased their working hours, and those who have since left work for a reason other than caring (e.g. own disability, retirement). Excludes primary carers who are not employed and did not work before commencing caring.

**Notes on Tables S5-S6**

Supplementary regression models explored whether the types, frequency and unmet need for assistance by the person being cared for were related to carers’ employment. Since types and frequency of formal assistance were strongly related for both male and female mental health carers, these variables were analysed separately. For each gender, two further models were conducted, replacing receipt of formal services by the person being cared for (yes/no) with: (a) whether the supported person has an unmet need for assistance, receipt of formal assistance with cognitive or emotional tasks, and receipt of formal assistance with other tasks (including assistance with household chores, meal preparation, property maintenance, reading or writing, communication, transport, health care, mobility, and self-care); and (b) whether the person being cared for has an unmet need for assistance, and frequency of formal assistance received (none, less than weekly, weekly or more).

Supplementary regression models for female carers found no significant association between female carers’ employment and the types, frequency, and unmet need for assistance by the person being cared (Tables A6 and A7).

Supplementary regression models for male carers found that the type and frequency of formal assistance received by the person with mental illness were related to male carers’ employment. Controlling for carer disability, relationship to the person supported, and disability level of that person, male mental health carers had lower odds of not being employed if the person cared for received formal assistance with cognitive or emotional tasks (vs. no assistance with cognitive or emotional tasks; AOR 0.35, 95% CI: 0.19-0.65, p=.001; Table A6), as well as if the person with mental illness received any type of formal assistance at least weekly (vs. no formal assistance; AOR 0.23, 95% CI: 0.08-0.68, p=.009; Table A7). Receipt of formal assistance with other practical or self-care tasks and unmet need for assistance by the person supported were not significantly related to employment status for male carers.

**Table S5.** Supplementary logistic regression analyses of association between recipient types of formal assistance, unmet need for assistance, other carer characteristics and not being employed for co-resident carers aged 15-64 years of adults with mental illness

| **Carer characteristic** | **Female carers (n=268)** | | |  | **Male carers (n=238)** | | |
| --- | --- | --- | --- | --- | --- | --- | --- |
|  | **% not employed  (95% CI)** | **AOR (95% CI)** | ***p*** |  | **% not employed  (95% CI)** | **AOR (95% CI)** | ***p*** |
| Age group |  |  |  |  |  | ns |  |
| 15-34 years | 64.7 (46.5-79.4) | 1.00 |  |  | 36.5 (23.0-52.4) |  |  |
| 35-54 years | 40.7 (31.1-51.1) | 0.31 (0.11-0.86) | **.03** |  | 24.0 (16.1-34.2) |  |  |
| 55-64 years | 65.9 (50.1-79.0) | 0.74 (0.24-2.34) | .61 |  | 38.2 (26.0-52.0) |  |  |
| Highest level of education^a^ |  |  |  |  |  | ns |  |
| Post-secondary degree/certificate | 36.6 (27.8-46.4) | 1.00 |  |  | 23.4 (16.4-32.4) |  |  |
| Senior secondary school (Year 11 or 12) | 67.9 (53.3-79.7) | 3.09 (1.41-6.74) | **.005** |  | 34.9 (21.6-51.0) |  |  |
| Junior secondary school (Year 10) or less | 74.1 (61.9-83.4) | 3.86 (1.59-9.39) | **.004** |  | 48.9 (33.8-64.2) |  |  |
| Carer’s own disability status |  |  |  |  |  |  |  |
| No disability | 40.3 (30.6-50.9) | 1.00 |  |  | 24.7 (16.6-35.0) | 1.00 |  |
| Has a disability | 68.0 (58.5-76.1) | 3.60 (1.68-7.69) | **.001** |  | 48.6 (32.6-64.9) | 3.76 (0.90-15.67) | .07 |
| Cares for their spouse/partner or adult child |  | ns |  |  |  |  |  |
| Cares for another relative/friend only | 65.9 (48.9-79.7) |  |  |  | 49.3 (34.7-64.0) | 1.00 |  |
| Cares for their partner/child | 49.7 (41.4-57.9) |  |  |  | 24.7 (18.7-31.9) | 0.38 (0.14-1.05) | .06 |
| Care recipient disability level |  |  |  |  |  |  |  |
| Moderate or less limitation in core activities | 42.1 (33.4-51.4) | 1.00 |  |  | 16.2 (8.5-28.6) | 1.00 |  |
| Profound or severe limitation in core activities | 64.1 (52.5-74.2) | 2.13 (1.02-4.43) | **.04** |  | 44.5 (34.0-55.5) | 3.87 (1.35-11.15) | **.01** |
| Care recipient(s) receipt of formal assistance with cognitive or emotional tasks |  | ns |  |  |  |  |  |
| Does not receive emotional assistance | 57.5 (46.3-68.1) |  |  |  | 44.1 (34.1-54.5) | 1.00 |  |
| Receives emotional assistance | 48.8 (38.1-59.7) |  |  |  | 19.8 (13.5-27.9) | 0.35 (0.19-0.65) | **.001** |

AOR – adjusted odds ratio; CI – confidence interval; ns – factor was not significantly related to employment at p>.10 and was not included in final model.

^a^ In Australia, Year 12 is the final year of secondary schooling, generally complete at age 17 or 18.

Notes: *p-*values in **bold** are significant at *p*<.05. The following variables were not significantly related to employment status and were removed from the final regression models: (1) for female mental health carers – marital status, rurality, country of birth, primary carer status, number of recipients of care, caring for their partner/child, care recipient unmet need for assistance, care recipient receipt of formal assistance with cognitive or emotional tasks, and care recipient receipt of formal assistance with other tasks; (2) for male mental health carers – age group, marital status, rurality, country of birth, education level, primary carer status, number of recipients of care, care recipient unmet need for assistance, and care recipient receipt of formal assistance with other tasks.

**Table S6.** Supplementary logistic regression analyses of association between recipient frequency of formal assistance, unmet need for assistance, other carer characteristics and not being employed for co-resident carers aged 15-64 years of adults with mental illness

| **Carer characteristic** | **Female carers (n=268)** | | |  | **Male carers (n=238)** | | |
| --- | --- | --- | --- | --- | --- | --- | --- |
|  | **% not employed  (95% CI)** | **AOR (95% CI)** | ***p*** |  | **% not employed  (95% CI)** | **AOR (95% CI)** | ***p*** |
| Age group |  |  |  |  |  | ns |  |
| 15-34 years | 64.7 (46.5-79.4) | 1.00 |  |  | 36.5 (23.0-52.4) |  |  |
| 35-54 years | 40.7 (31.1-51.1) | 0.31 (0.11-0.86) | **.03** |  | 24.0 (16.1-34.2) |  |  |
| 55-64 years | 65.9 (50.1-79.0) | 0.74 (0.24-2.34) | .61 |  | 38.2 (26.0-52.0) |  |  |
| Highest level of education^a^ |  |  |  |  |  | ns |  |
| Post-secondary degree/certificate | 36.6 (27.8-46.4) | 1.00 |  |  | 23.4 (16.4-32.4) |  |  |
| Senior secondary school (Year 11 or 12) | 67.9 (53.3-79.7) | 3.09 (1.41-6.74) | **.005** |  | 34.9 (21.6-51.0) |  |  |
| Junior secondary school (Year 10) or less | 74.1 (61.9-83.4) | 3.86 (1.59-9.39) | **.004** |  | 48.9 (33.8-64.2) |  |  |
| Carer’s own disability status |  |  |  |  |  |  |  |
| No disability | 40.3 (30.6-50.9) | 1.00 |  |  | 24.7 (16.6-35.0) | 1.00 |  |
| Has a disability | 68.0 (58.5-76.1) | 3.60 (1.68-7.69) | **.001** |  | 48.6 (32.6-64.9) | 3.99 (1.02-15.54) | **.046** |
| Cares for their spouse/partner or adult child |  | ns |  |  |  |  |  |
| Cares for another relative/friend only | 65.9 (48.9-79.7) |  |  |  | 49.3 (34.7-64.0) | 1.00 |  |
| Cares for their partner/child | 49.7 (41.4-57.9) |  |  |  | 24.7 (18.7-31.9) | 0.36 (0.12-1.12) | .08 |
| Care recipient disability level |  |  |  |  |  |  |  |
| Moderate or less limitation in core activities | 42.1 (33.4-51.4) | 1.00 |  |  | 16.2 (8.5-28.6) | 1.00 |  |
| Profound or severe limitation in core activities | 64.1 (52.5-74.2) | 2.13 (1.02-4.43) | **.04** |  | 44.5 (34.0-55.5) | 4.39 (1.43-13.48) | **.01** |
| Care recipient(s) frequency of formal services |  | ns |  |  |  |  |  |
| Does not receive services | 55.3 (42.7-67.3) |  |  |  | 44.3 (33.0-56.1) | 1.00 |  |
| Receives services less than weekly | 48.8 (36.6-61.2) |  |  |  | 25.2 (17.1-35.5) | 0.50 (0.23-1.09) | .08 |
| Receives services weekly or more | 57.5 (41.7-71.9) |  |  |  | 18.2 (8.9-33.4) | 0.23 (0.08-0.68) | **.009** |

AOR – adjusted odds ratio; CI – confidence interval; ns – factor was not significantly related to employment at p>.10 and was not included in final model.

^a^ In Australia, Year 12 is the final year of secondary schooling, generally complete at age 17 or 18.

Notes: *p-*values in **bold** are significant at *p*<.05. The following variables were not significantly related to employment status and were removed from the final regression models: (1) for female mental health carers – marital status, rurality, country of birth, primary carer status, number of recipients of care, caring for their partner/child, care recipient unmet need for assistance, and care recipient frequency of formal assistance; (2) for male mental health carers – age group, marital status, rurality, country of birth, education level, primary carer status, number of recipients of care, and care recipient unmet need for assistance.
